# Multifunctional PEGylated Niosomal Nanoparticle-Loaded Herbal Drugs as a Novel Nano-Radiosensitizer and Stimuli-Sensitive Nanocarrier for Synergistic Cancer Therapy

**DOI:** 10.3389/fbioe.2022.917368

**Published:** 2022-08-15

**Authors:** Saeid Afereydoon, Fateme Haghiralsadat, Nima Hamzian, Ali Shams, Mahdie Hemati, Seyed Morteza Naghib, Masoud Shabani, Behrouz Zandieh-doulabi, Davood Tofighi

**Affiliations:** ^1^ Department of Medical Physics, School of Medicine, Shahid Sadoughi University of Medical Sciences, Yazd, Iran; ^2^ Department of Advanced Medical Sciences and Technologies, School of Paramedicine, Shahid Sadoughi University of Medical Sciences, Yazd, Iran; ^3^ Medical Nanotechnology and Tissue Engineering Research Center, Yazd Reproductive Sciences Institute, Shahid Sadoughi University of Medical Sciences, Yazd, Iran; ^4^ Department of Immunology, School of Medicine, Shahid Sadoughi University of Medical Sciences, Yazd, Iran; ^5^ Department of Clinical Biochemistry, Faculty of Medicine, Shahid Sadoughi University of Medical Sciences, Yazd, Iran; ^6^ Nanotechnology Department, School of Advanced Technologies, Iran University of Science and Technology (IUST), Tehran, Iran; ^7^ Biomaterials and Tissue Engineering Department, Breast Cancer Research Center, Motamed Cancer Institute, ACECR, Tehran, Iran; ^8^ Department of Radiation Oncology, School of Medicine, Shahid Sadoughi University of Medical Sciences, Yazd, Iran; ^9^ Department of Oral Cell Biology, Academic Centre for Dentistry Amsterdam (ACTA), University of Amsterdam and Vrije Universiteit Amsterdam, Amsterdam Movement Sciences, Amsterdam, Netherlands; ^10^ Epidemiology and Research Design Support (BERD), Clinical and Translational Science Center, Department of Psychology, University of New Mexico, Albuquerque, NM, United States

**Keywords:** niosome nanoparticles, irradiation, curcumin, radiosensitizing, breast cancer

## Abstract

Nowadays, radiotherapy is one of the most effective treatments for breast cancer. In order to overcome the radioresistance of cancer cells, radio-sensitizing agents can be used combined with irradiation to increase the therapeutic efficiency. Curcumin can enhance the radiosensitivity of cancer cells and decrease their viability by the accumulation of these cells in the G2 phase. The encapsulation of curcumin in a nanoniosomal delivery system increases aqueous solubility and bioavailability, resulting in increased radio sensitivity. The present study aimed to enhance the radio-sensitizing effect of the curcumin-containing nanoniosome (Cur-Nio) when combined with irradiation. Thus, curcumin (0.5 mg ml^−1^) was loaded on a PEGylated nanoniosome containing Tween 60, cholesterol, DOTAP, and 1,2-distearoyl-sn-glycero-3-phosphoethanolamine-poly(ethylene glycol) (DSPE-PEG) (at ratios of 70:30:10:5, respectively) by the thin-film hydration method. The particle size, zeta potential, entrapment efficiency, and drug-release rate of formulated nanoniosomes were determined. In order to assess cytotoxicity and apoptosis, different doses of irradiation along with various concentrations of free curcumin and Cur-Nio (single or in combination with irradiation) were treated with breast cancer cells. The particle size and zeta potential of Cur-Nio were reported to be 117.5 nm and −15.1 mV, respectively. The entrapment efficiency (EE%) and loading capacities were 72.3% and 6.68%, respectively. The drug-release rate during 6 h was 65.9%. Cell survival in the presence of curcumin at doses of 1 and 3 Gy showed a significant reduction compared with cells irradiated at 48 h and 72 h (*p* < 0.000). Also, the rate of cytotoxicity and apoptosis was significantly higher in cells treated with the combination of curcumin-containing nanoniosomes and irradiation in comparison with those treated with free curcumin. These findings indicate that the efficacy of pre-treatment with Cur-Nio as a radiosensitizer during radiotherapy enhances irradiation-induced breast cancer cell apoptosis and is a useful strategy to increase the effectiveness of breast cancer therapy.

## Introduction

Irradiation therapy (Yi et al., 2021; Zhao et al., 2021), chemotherapy (Jiang et al., 2021; Li et al., 2021), surgery (Cui et al., 2021), and hormone therapy (Im et al., 2019) are the most common therapies used for the treatment of breast cancer. Nowadays, radiotherapy is used as first-line therapy in patients with breast cancer depending on the grade of the tumor used alone or along with surgery or chemotherapy (Garg et al., 2005). The main goal of radiotherapy is established on reducing the side effects on normal tissues by minimizing the irradiation dose while providing the maximum efficiency to induce cell death in tumors (Crokart et al., 2005). The combination of chemotherapeutic agents with some chemical radio-sensitizers increases the rate of targeted therapies (Goel & Aggarwal, 2010). The use of synthetic radio-sensitizers can cause nausea, vomiting, hypotension, and allergic reactions due to their toxicity, even in daily prescribed doses. Therefore, it is still necessary to identify new, non-toxic, effective, and suitable compounds to increase radiation sensitivity. Plants, as non-toxic (or low-toxic) agents, are readily available and appear to be the ideal low-cost solution. Curcumin is a potent chemosensitizer known as a radio-sensitizing agent in various types of cancer. It seems that the radiosensitizing effects of curcumin are mediated *via* the inhibition of NF-κB activity in colon cancer (Kunnumakkara et al., 2008; Hamzian et al., 2017; Hamzian et al., 2019), reducing the signaling pathways of AP-1 and NF-κB in brain cancer (Dhandapani et al., 2007), downregulation of Bcl-2 and MDM2 proteins in prostate cancer (Li et al., 2007; Ghaffari et al., 2021), reduction of extracellular regulated kinases (ERK1 and 2), overexpression of the epidermal growth factor receptor (EGFR) in skin cancer (Park & Lee, 2007), and suppression of cells in the G2/M cell cycle phase in squamous cell carcinoma (Khafif et al., 2005). Overall, these investigations suggest that the combination of curcumin with irradiation is a potential treatment to increase the effectiveness of treatment regimens on breast cancers. On the other hand, curcumin is hydrophobic and insoluble in water and is poorly absorbed by the intestine, leading to the limited bioavailability and concentration of this compound in the serum after oral administration (Liu et al., 2016; Abtahi N. A. et al., 2021; Yaghoubi et al., 2021). In order to overcome these challenges, nanotechnology has provided a multifunctional nano-delivery system, which acts as a drug carrier, improving the *in vivo* stability, bioavailability, and biodistribution of curcumin (Sim & Wong, 2021). Nanoparticles may consist of silver, gold, and iron oxide nanoparticles (metallic nanoparticles) or could be made of liposome, noisome, and micelle (lipid nanoparticles). These nanoparticles are able to be loaded with different hydrophilic and hydrophobic compounds. Metallic nanoparticles play an important role in radiosensitization as a result of having a high atomic number (*Z*) of metals, photoelectric absorption, and the production of hydroxyl radicals (Butterworth et al., 2013; Siddique & Chow, 2020). Encapsulation of curcumin in the form of nanoparticles at the nanoscale size leads to increased biocompatibility, cellular internalization, and water solubility, whereas decreases the side effects of the loaded compound in the process of cancer treatment. Several studies have demonstrated the radiosensitivity potential of curcumin encapsulated in metallic and lipidic nanoparticles (Minafra et al., 2019; Gao et al., 2022); however, the impact of curcumin-containing nanoniosomes on the radiosensitivity of cancer cells has not been evaluated. Niosomes have considerable advantages, including acceptable stability, long-lasting stability in the bloodstream, small size, high encapsulation efficacy, no special condition for the storage process, and low cast. In this study, to boost pharmacological properties, curcumin was encapsulated in the form of nanoniosomes, which are bilayer non-ionic surfactant-based vehicles. These particles are stable, non-toxic, and biodegradable at the nanoscale size and could be employed as a novel drug delivery system (Kalantari et al., 2019). Tween 60, with hydrophilic-lipophilic balance (HLB) of 14.9, is not appropriate as the main surfactant for the formulation of curcumin-containing nanoniosomes. So the presence of cholesterol in niosomal compounds provides high physical stability for these nanoparticles (Abtahi N. A. et al., 2021).

**FIGURE 1:**
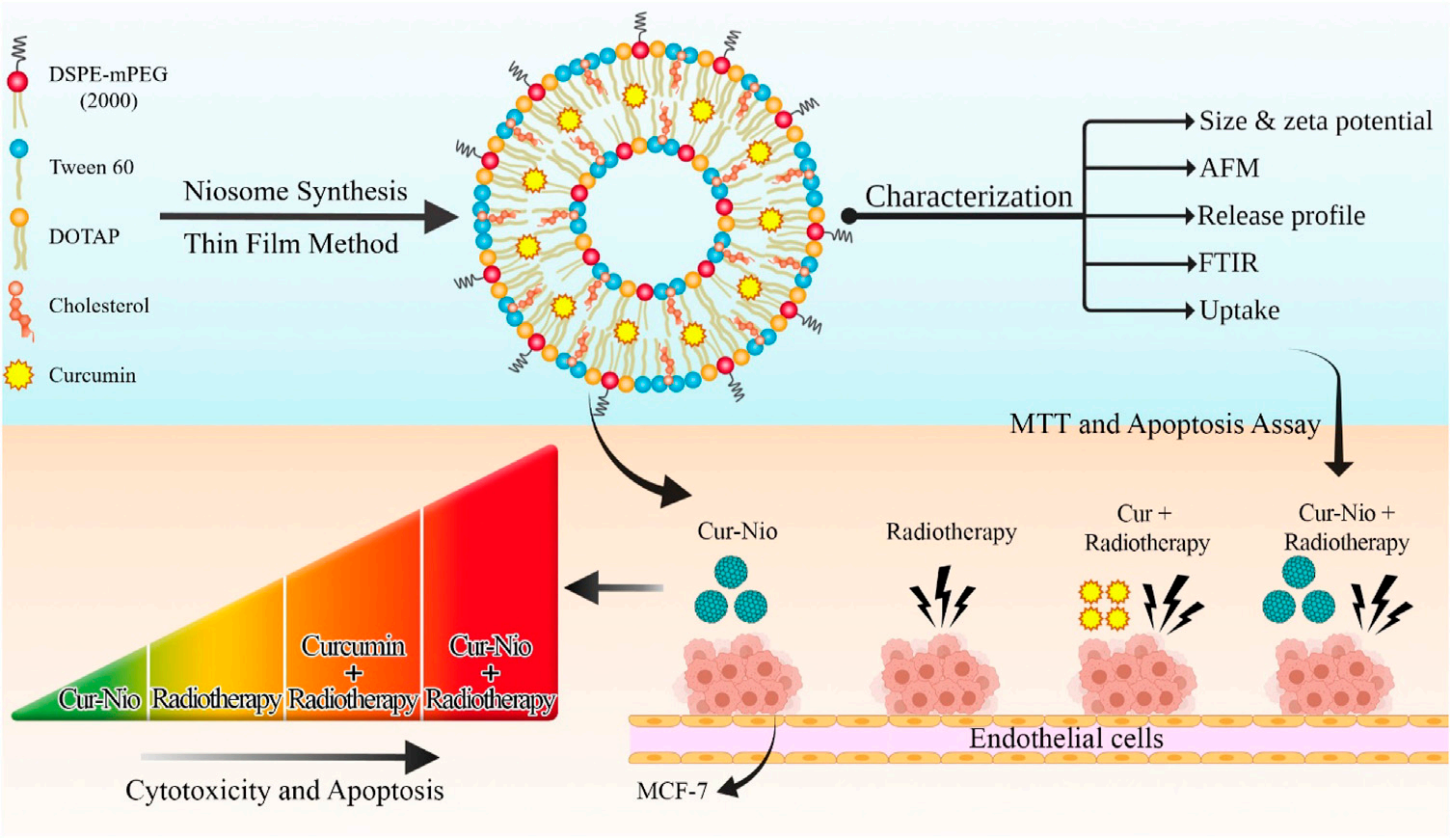
Schematic view of synthesizing the PEGylated niosomal nanoformulation based on Tween 60, cholesterol, DOTAP, and DSPE-PEG at ratios of 70:30:10:5, respectively. The process of curcumin encapsulation was performed by the thin-film hydration method and then used in combination with radiotherapy.

The current study evaluated the radiosensitizing effect of nanoniosomes containing curcumin. For this purpose, we developed cationic PEGylated nanoniosome-containing curcumin with high entrapment efficiency (EE%), nanoscale size, and a controlled release profile. In our study, the breast cancer cell line (MCF-7) was irradiated with ionizing beams in the presence or absence of Cur-Nio. The MTT assay and flow cytometry were applied to evaluate cell viability and apoptosis between cells treated with free curcumin and cationic PEGylated nanoniosome-containing curcumin (Figure 1).

## Materials and Methods

The thin-film hydration method was used to synthesize nanoliposomes containing curcumin with desired vesicle size, high entrapment efficiency, and the controlled release rate. Cholesterol (Sigma-Aldrich, United States), DOTAP, Tween 60 (DaeJung Chemicals & Metals, South Korea), and DSPE-PEG2000 (Ludwigshafen, Germany) were dissolved in chloroform. Curcumin (Sigma-Aldrich, United States) at a concentration of 0.5 mg/ml was dissolved in methanol and poured into a container. In order to evaluate cellular uptake, 0.1% M of the fluorescent label Dil (Sigma, United States) was added to the lipid phase. The organic solvent was eliminated by using a rotary evaporator (Heidolph, Germany) at 45°C to form a dried lipid thin film. After that, it was hydrated with a suitable volume of phosphate-buffered saline for 60 min at 60°C. Sonication was performed using a microtip probe sonicator (model UP200St, Hielscher Ultrasonics GmbH, Germany) on ice for 30 min to reduce the particle size. Finally, a dialysis membrane (M.W. = 12 kDa, Sigma-Aldrich, United States) was utilized to separate the free drug from niosomal vesicles.

### Particle Size and Zeta-Potential Values

The range of particle size distribution, as well as the peak particle size, was assessed by the dynamic light scattering (DLS) technique (the Brookhaven Instruments Corp). Nanoniosomes were diluted in distilled water and measured at an angle of 90°, and the laser light was emitted at a wavelength of 657 nm at 25 ± 1°C. Zeta potential values (ζ-potential) were evaluated at 25 ± 1°C by using a zeta-sizer instrument (the Brookhaven Instruments Corp).

### Drug-Loading Capacity and Entrapment Efficiency Determination

The standard concentrations of curcumin were prepared by dissolving a serial concentration of curcumin in isopropanol, and their absorption was detected by using a UV-VIS spectrophotometer (model T80+, P.G. Instruments, United Kingdom) at 429 nm. Also, nanoniosome-containing curcumin was dissolved in isopropanol with a certain dilution, and its absorption was obtained using a spectrophotometer at 429 nm. The entrapment efficiency of curcumin in nanoniosome was calculated based on the low formula using a standard curve:

EE % = drug concentration in nanocarrier (mg.ml^−1^)/total drug (mg.ml^−1^) ×100.

The loading capacity of curcumin was evaluated by using a UV-Vis spectrophotometer (Perkin Elmer Germany) at 429 nm as follows:

LC % = mass of drug in nanocarrier (mg)/mass of nanocarrier (mg)× 100.

### *In Vitro* Drug Release Study

The *in vitro* release profile of nanoniosome-containing curcumin was measured using a dialysis bag with a molecular weight cut-off of 12,000–14,000 kDa against PBS while being stirred on a magnetic stirrer for 72 h at 37°C (pH = 7.4). In order to calculate the drug-released profile at different times, the dialysis medium was collected and immediately exchanged for an equal volume of the fresh PBS. The absorbance of specimens was measured at 429 nm using a UV–VIS spectrophotometer. Based on the total concentration of niosomal drugs, the release percentage at different time intervals was calculated.

### Fourier Transform Infrared Spectroscopy

The characterization of functional groups and chemical interactions between curcumin and nanoniosome components was analyzed by Fourier transform infrared (FTIR) spectroscopy (Model 8300, Shimadzu Corporation, Tokyo, Japan). The FTIR spectrum was analyzed at the wavelength range of 400–4000 cm^−1^ in samples dispersed in KBr pellets.

### Irradiation Procedures

The source-to-axis distance (SAD) irradiations were conducted in a 20 × 20 cm^2^ field, with the gantry angle being 180 and 5 cm deep in solid phantom equivalent to tissue. The monitor unit was performed by Panther three-dimensional treatment design software version 5.2 (Prowess-USA). In order to optimize the dosimetry condition, central wells of the plate were used for cell culture as much as possible, and the culture medium was added to empty wells. Then, irradiation was performed using X-rays of a linear accelerator photon beam (Compact-Electa, England) to assess the irradiation efficacy, thereby creating a dose-dependent curve (1, 3, 5, and 7 Gy).

### Cell Culture

Human breast cancer (MCF-7), lung cancer (A549), osteosarcoma (Saos2), and prostate cancer (PC3) cell lines were purchased from the Pasteur Institute, Tehran, Iran. They were grown in DMEM (Dulbecco’s modified Eagle’s medium, Gibco, Dublin, Ireland) supplemented with 10% fetal bovine serum (FBS Gibco) and 100 U/ml penicillin-streptomycin (Gibco) in a 5% CO_2_ incubator at 37°C.

### Cellular Uptake of Nanoniosome–Containing Curcumin

In order to determine and compare the cellular uptake behavior of Cur-Nio and free curcumin, MCF7, A549, Saos2, and PC3 cells were seeded on to 6-well plates at a density of 1.5 × 10^5^ cells/well. Then, the cells were incubated with free curcumin, Cur-Nio (20 μg ml^−1^), and blank nanoniosomes. After 3 h, cells were washed twice with fresh cold PBS (pH 7.4) and fixed with the 96% ethanol solution. The nuclei were counterstained with DAPI (0.15 μg ml^−1^, Thermo Fisher Scientific, United States) and visualized under a fluorescence microscope (BX61, Olympus, Japan).

### Cytotoxicity Assay

In order to examine whether curcumin could be considered a radio-sensitizing agent, the cytotoxicity effects of irradiation with or without curcumin and Cur-Nio on MCF7 cells were evaluated. The inhibitory concentration (IC10) of drugs, a concentration without obvious drug toxicity, and the lethal dose (LD50) of radiation, as a minimum dose with the highest efficacy and no toxicity, were determined. To this aim, 10^4^ cells were seeded onto a 96-well plate with five replicates for each treatment. After 24 h, the different doses of irradiation (1, 3, 5, and 7 Gy) or Cur-Nio at different concentrations (2, 5, 10, 20, 40, 80, and 100 μg ml^−1^) were treated with cancer cell lines. For combinational therapy, Cur-Nio at a dose of 20 μg ml^−1^ (IC10) or free curcumin was treated with cell lines for 24 h before being irradiated at doses of 1 and 3 Gy (LD50). Untreated cells were considered the control cells. Finally, after 48 and 72 h of incubation, the cell culture media were removed, and 100 μl of the MTT solution [10 μl of the MTT reagent (0.5 mg/ml) + 90 μl culture media] was added to each well and incubated for 4 h at room temperature. The formazan crystals were dissolved in 150 µl DMSO. The absorbance of each well was read by using a spectrophotometer (Synergy HTX, Bio-Tek, Winooski, VT) instrument at a wavelength of 570 nm. The cell viability rate of different treatments was calculated compared with the control group.

### Apoptosis Analysis

In order to examine the effect of curcumin-loaded nanoniosome and irradiation, either alone or in combination, Annexin-V and propidium iodide assays were carried out. In brief, in each well, 4×10^4^ MCF-7 cells were seeded onto a 24-well microplate. After 24 h, cells were divided into nine groups. In group 1, MCF-7 cells were left without being exposed to any treatments; MCF-7 cells in groups 2 and 3 were irradiated at doses of 1 and 3 Gy, respectively. In groups 4 and 5, MCF-7 cells were incubated with curcumin and Cur-Nio at a concentration of 20 μg/ml, respectively. In groups 6 and 7, MCF-7 cells were treated with curcumin and Cur-Nio at a concentration of 20 μg/ml and irradiated at a dose of 1 Gy. In groups 8 and 9, MCF-7 cells were treated with curcumin and Cur-Nio at a concentration of 20 μg/ml and irradiated at a dose of 3 Gy, following pretreatment. After 24 and 72 h, the total cells were isolated by 0.05% trypsin/EDTA, centrifuged, and rinsed in 100 µl binding buffer. Then, the cells were suspended with Annexin V-FITC and propidium iodide (P.I.) as fluorescent dyes and incubated in a dark place without light for 20 min. The samples were analyzed by using a flow cytometer (B.D. Biosciences, United States).

### Statistical Analysis

The results were analyzed by GraphPad Prism version 8 software, and the obtained values were represented as the means and standard deviation (mean ± SD). The independent t-test was applied to compare two groups, while a one-way analysis of variance (ANOVA) was used to compare multiple groups. A *p*-value of less than 0.05 was considered a statistically significant difference.

## Results

### Characterization of Synthesized Nanoparticles

**FIGURE 2:**
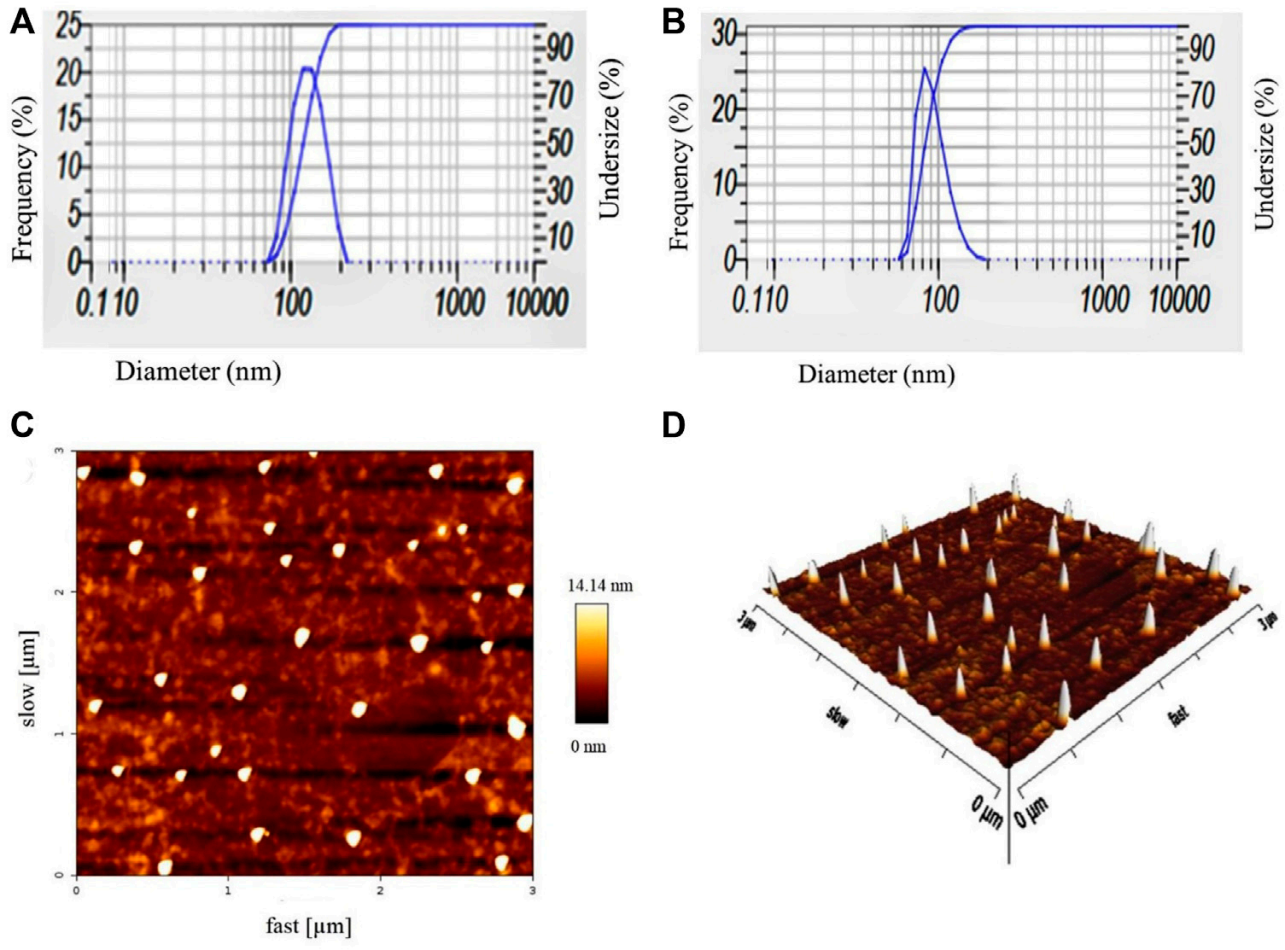
Particle size distribution and zeta potential values of blank nanoniosomes **(A)** and Cur-Nio **(B)**. Two-dimensional **(C)** and three-dimensional **(D)** AFM images of Cur-Nio.

**FIGURE 3:**
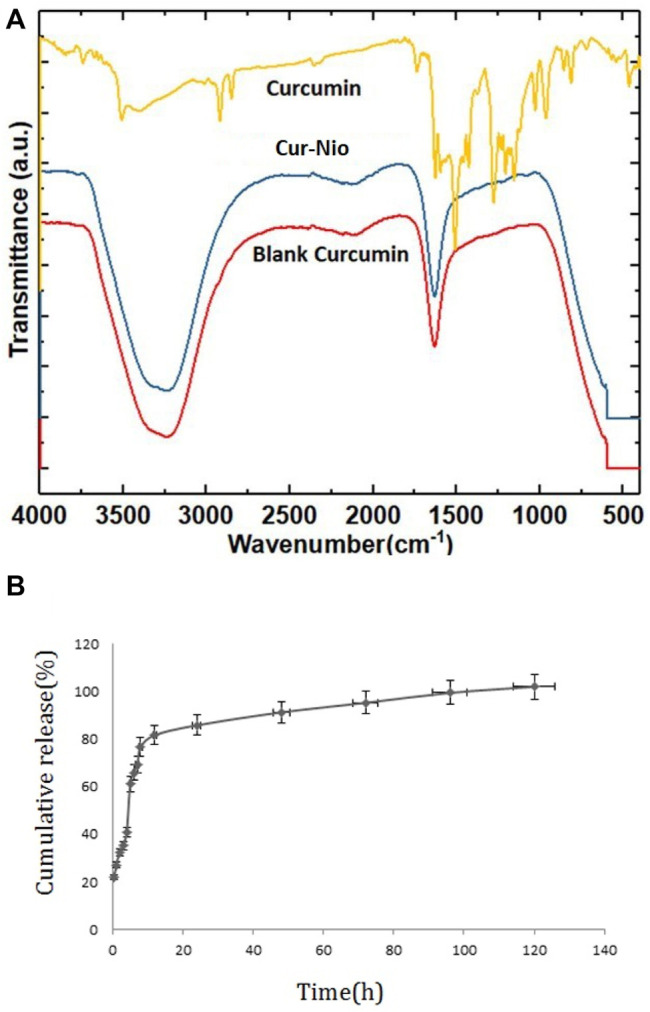
FTIR spectra; a comparison of free curcumin, Cur-Nio, and blank nanoniosome **(A)**. The *in vitro* release profile of curcumin from the nanoniosome system **(B)**.

The DLS analysis determined the mean hydrodynamic diameter, polydispersity index (PDI) of blank nanoniosomes, and Cur-Nio. As shown in Table 1 and Figures 2A,B, blank nanoniosomes and Cur-Nio exhibit average diameters of 117.5 and 108.7 nm and PDI values of 0.403 and 0.305, respectively. The zeta potential values of the blank nanoniosomes and Cur-Nio are shown in Figure 2. The great extent of the negative zeta potential and anionic nanoparticles enhanced the potential stability of the nanosystem solution. The two-dimensional, three-dimensional, and surface morphology of Cur-Nio were characterized by atomic force microscopy (AFM) (Figures 2C,D). All results were in accordance with the findings of the DLS analysis. The chemical structure of compounds in the synthesized nanoniosome and curcumin, as well as the molecular interaction of curcumin with a lipid membrane of nanoniosomes, was applied by FTIR spectroscopy. Figure 3A illustrates the FTIR spectra of the free curcumin. The absorption bands at 3510 cm^−1^ were assigned for the O–H stretching bond; the absorption peak at 1509 cm^−1^ corresponds to the aromatic ring, C=C stretching, and C=O stretching bonds at 1155 cm^−1^. The absorption peak at 1155cm^−1^ was assigned to the C–H stretching bond in the curcumin molecule. The blank nanoniosome exhibits various peaks of FTIR patterns for DOTAP, Tween-60, cholesterol, and DSPE-mPEG in a range of 4000–500 cm^−1^ (Figure 3A). The peak at 3243 cm^−1^ was assigned to the existence of the O–H stretching bond in phenols and N–H stretching in 2°-amines of cholesterol and Tween-60. The carbonyl group in Tween-60, DOTAP, and DSPE-mPEG demonstrated a strong peak at 1632 cm^−1^, corresponding to the C=O stretching vibration.

**TABLE 1:** Characterization of blank nanoniosomes and Cur-Nio.

Formula	Lipid (molar ratio%) cholestrol: Tween 60	DOTAP (%)	DSPE-PEG (%)	Size (nm)	PDI	Zeta potential (mV)	EE (%)	LC (%)	Release (6 h)%
Blank nanoniosome	30:70	10	5	108.7	0.305	-20.1	-	**-**	**-**
Cur-Nio	30:70	10	5	117.5	0.423	-15.1	72	6.68	65.9%

Additional drug bands were not visible in the FTIR diagram of nanoniosomes. This phenomenon results from the complete encapsulation of curcumin in niosomal spherical nanocarriers, and therefore, the functional groups of these compounds are masked. On the other hand, long fatty acid chains with abundant hydrocarbon functional groups overlap and eliminate other functional groups. Curcumin encapsulated in nanocarrier membranes did not form any chemical interactions between curcumin and PEGylated niosomes. Also, the loading of curcumin on PEGylated niosomes did not affect its structural characterization. The *in vitro* curcumin release from nanoniosomes was evaluated using the dialysis bag method against the PBS buffer at 37°C. As exhibited in Figure 3B, the release of loaded drugs was 65.9% after 6 h. The cumulative release profile of curcumin showed a biphasic pattern, the first phase with an initial burst release period followed by a slower release rate in the second phase.

### Nanoniosomal Curcumin Cellular Uptake Experiments

**FIGURE 4:**
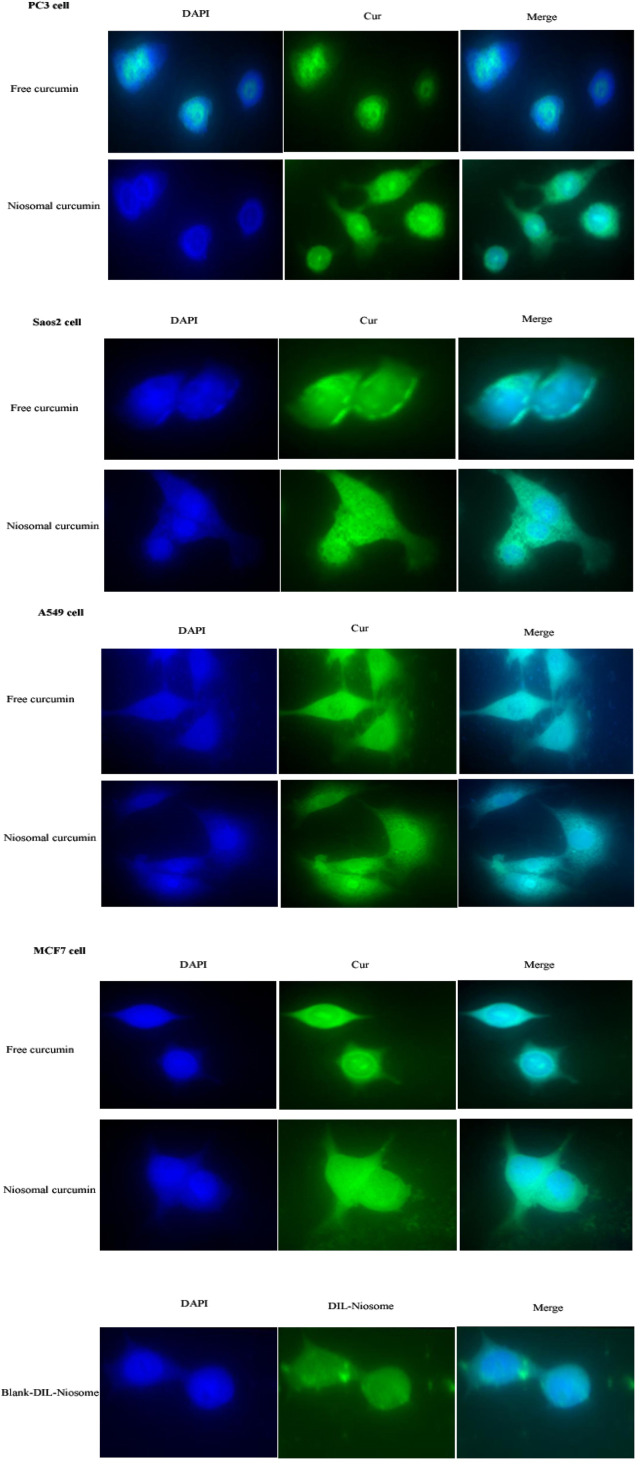
(Continued). Cellular uptake of the free curcumin, Cur-Nio, and blank-DIL-niosomes on PC3, SaOs_2_, A549, and MCF7 cell lines (×60 magnification).

The cellular uptake of PEGylated nanoniosomes was assessed by fluorescence microscopy to evaluate and compare the penetration and localization of the free curcumin and Cur-Nio. The fluorescence images of niosome uptake were taken after 3 h of continuous exposure of niosomes to the cells. As depicted in Figure 4, the intensity of green and cyan colors of nanoniosomal curcumin in cancer cells was much higher than that of the free curcumin. These findings exhibit that the amounts of Cur-Nio formulations inside cancer cells were significantly higher than those of the free curcumin. These results were in accordance with cytotoxicity and radio-sensitization results. Also, Figure 4 represents the successful internalization of DIL-labeled niosomes into cells.

### Cytotoxicity of the Modified Curcumin-Loaded Nanoniosomes

**FIGURE 5:**
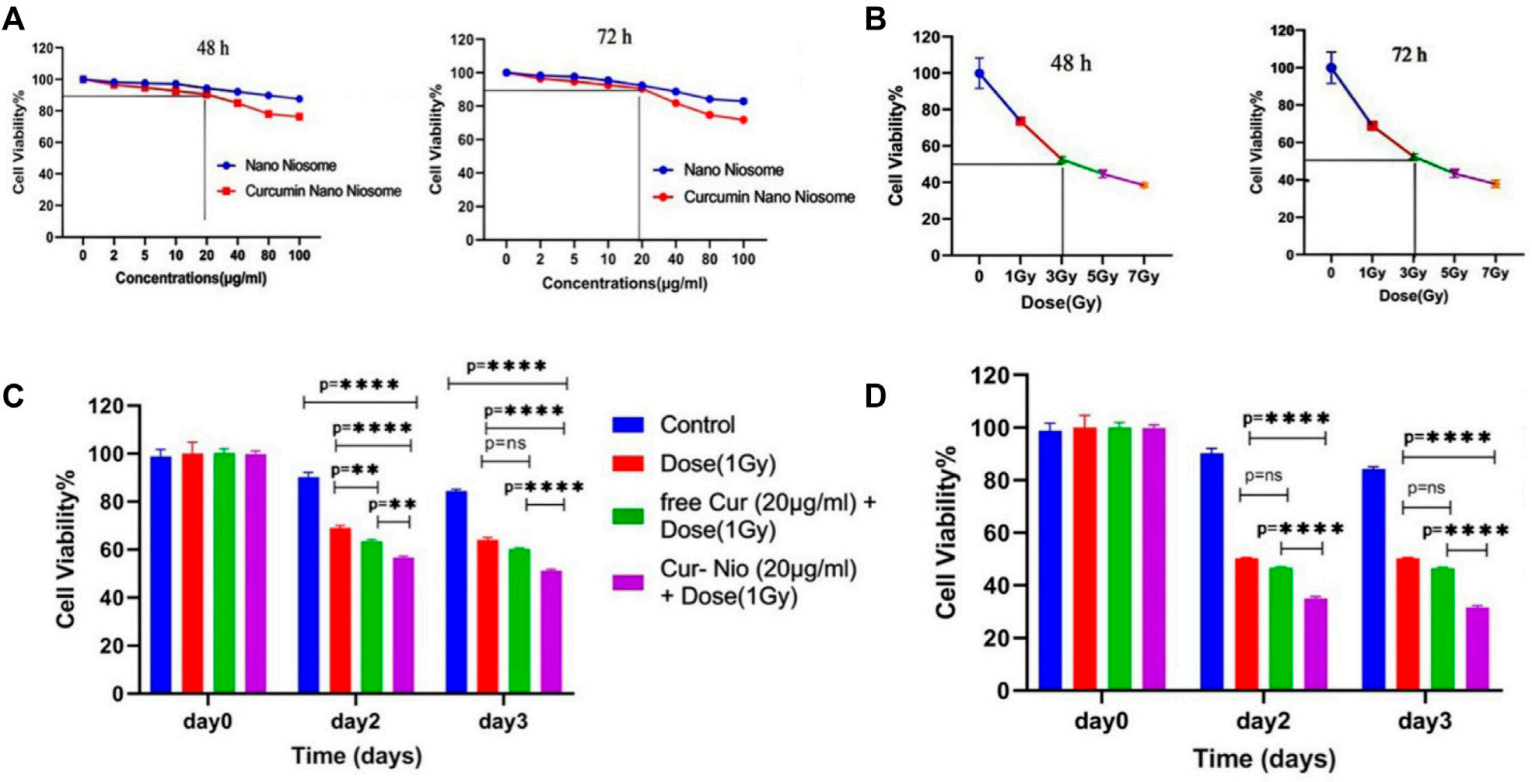
Viability of MCF7 cells treated with blank nanoniosomes and Cur-Nio at 48 and 72 h **(A)**. Dose-response curves of MCF7 cells that were exposed to increasing irradiation doses at 48 and 72 h **(B)**. The viability of MCF7 cells treated with irradiation with or without 20 μg ml^−1^ of free-Cur **(C)** and Cur-Nio. **(D)** NS stands for “not statistically significant;” **p < 0.01 and ****p < 0.0001.

In order to examine whether there was a radio-sensitive effect in the use of free curcumin or Cur-Nio along with irradiation, the MTT assay was conducted. The LD50 value was calculated from the ir radiation dose-response curves acquired by the increase in concentrations of irradiation, using a second-order polynomial fitting analysis. The MTT assay exhibited that blank nanoniosomes had no significant cytotoxic effects on MCF-7 cells at concentrations used (Figure 5A **)**. In order to assay the IC10 value of Cur-Nio and LD50 of irradiation against MCF7 cells, a dose-response experiment was performed. The IC10 value of Cur-Nio and the LD50 value of irradiation were reported to be 20 μg ml^−1^ (Figure 5A) and 3 Gy (Figure 5B), respectively. As shown in Figures 5C,d, cell death and radio-sensitization effect were increased, following irradiation with Cur-Nio compared with exposure to 1 and 3 Gy (*p* < 0.05) alone. This result demonstrates that curcumin plays a major role in radio-sensitizing and irradiation-induced cell death. In the combined treatments, the level of cell viability is always significantly lower than that observed in samples exposed to irradiation alone, indicating the efficacy of the nano-delivery system in the potent induction of radio-sensitization. Also, a comparison of the dose enhancement ratios (DERs) of the free curcumin and Cur-Nio was calculated after 48 and 72 h at a dose of 3Gy irradiation. As illustrated in Tables 2, 3, such a ratio was 1.5 folds higher in Cur-Nio than that of the free curcumin after 48 and 72 h.

**TABLE 2:** Comparison of dose enhancement ratios (DER) between the free curcumin and Cur-Nio at a single dose of 3 Gy after 48 h.

Therapeutic group	Dose (3 Gy)	*p*-value	DER (3 Gy)
Control	52.53	-	-
Free curcumin	46.58	0.0003	1.12
Cur-Nio	34.96	≤0.0001	1.50

**TABLE 3:** Comparison of dose enhancement ratios (DER) between the free curcumin and Cur-Nio at a single dose of 3 Gy after 72 h.

Therapeutic group	Dose (3 Gy)	*p*-value	DER (3 Gy)
Control	50.14	-	-
Free curcumin	46.37	0.0003	1.07
Cur-Nio	31.66	≤0.0001	1.57

### Apoptosis Assay

**FIGURE 6:**
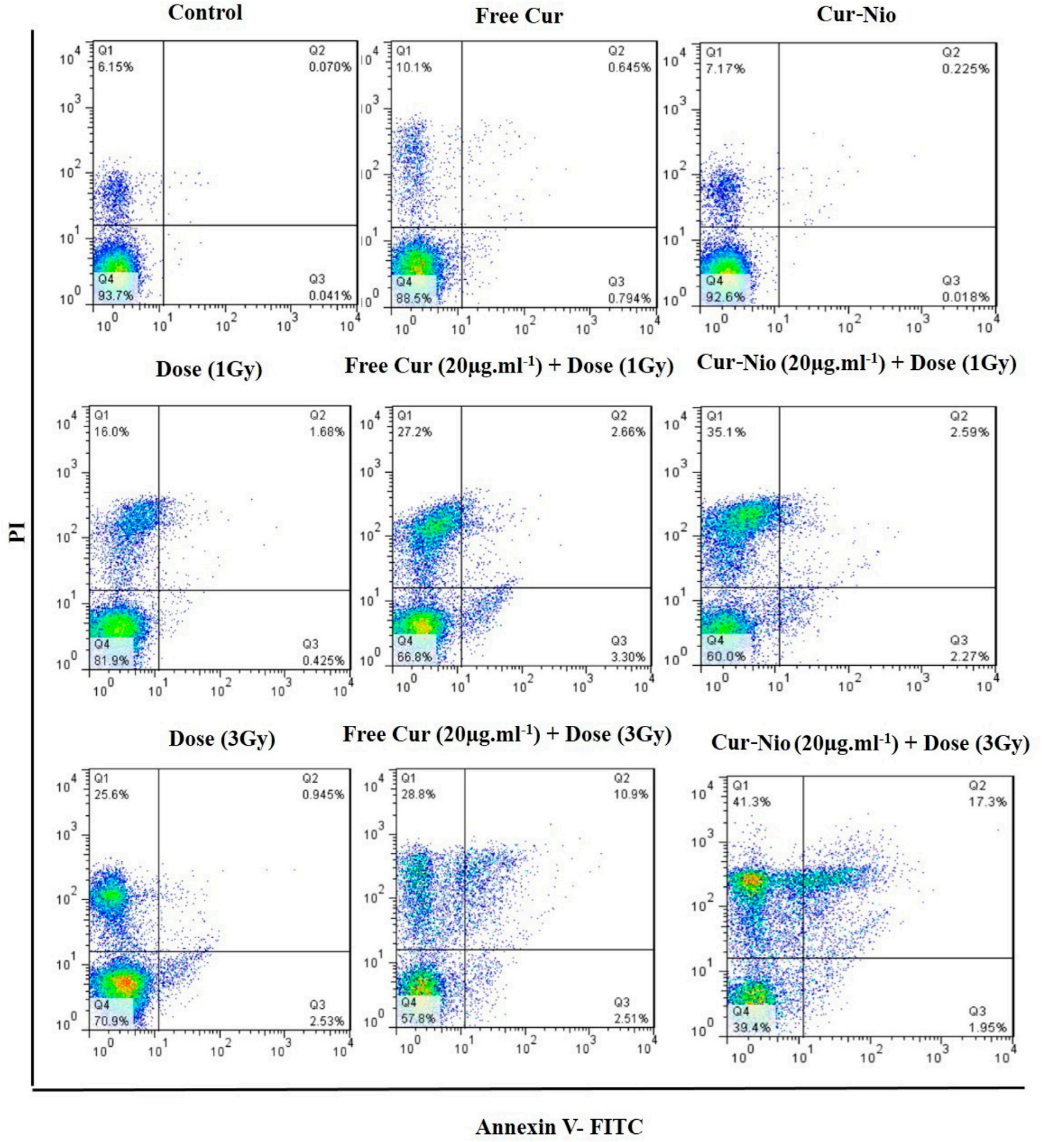
Apoptosis assay using flow cytometry, following treatment of cells at 48 h.

**FIGURE 7:**
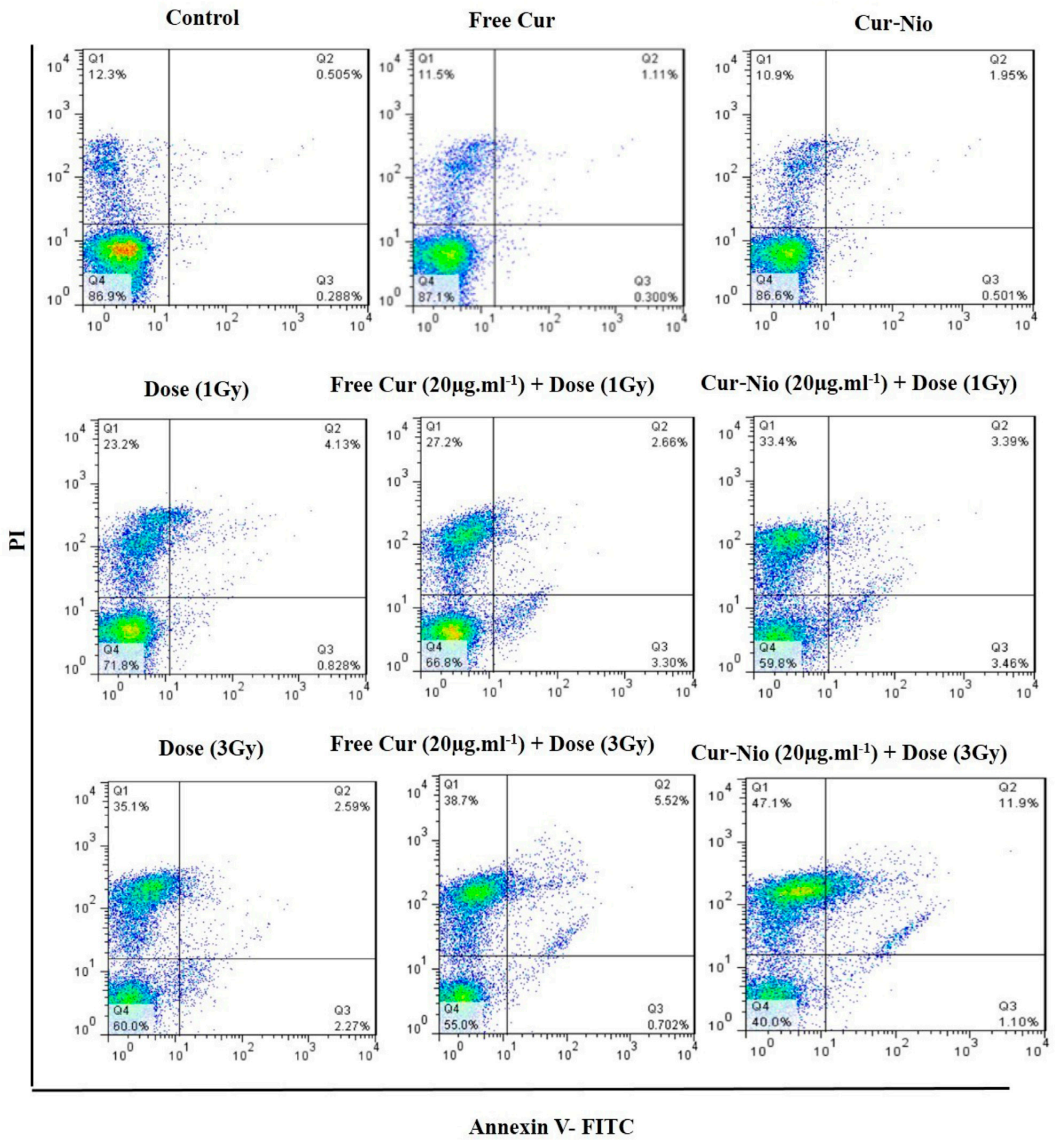
Apoptosis assay using flow cytometry, following treatment of cells at 72 h.

**FIGURE 8:**
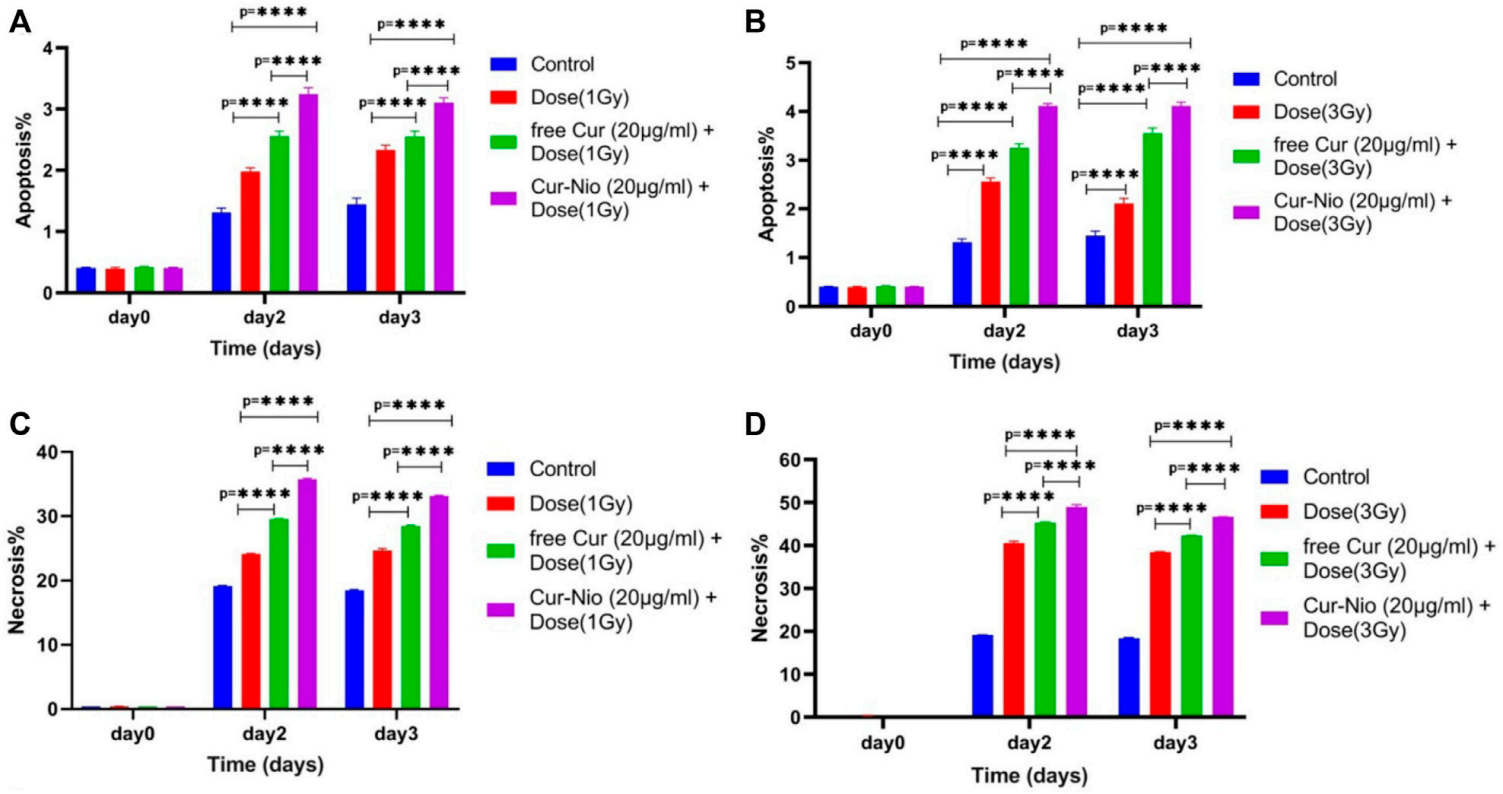
Apoptosis rate of MCF7 cells treated with irradiation at doses of **(A)** 1 Gy and **(B)** 3 Gy with or without 20 μg ml^−1^ of the free curcumin and Cur-Nio (****p < 0.0001). The necrosis rate of MCF7 cells treated with irradiation doses of **(A)** 1 Gy and **(B)** 3 Gy with or without 20 μg ml^−1^ of the free curcumin and Cur-Nio (****p < 0.0001).

Flow cytometry was used to evaluate the percentage of apoptosis and necrosis in MCF7 cells exposed to irradiation using a 6-MV linear accelerator at doses of 1 and 3 Gy alone or in combination with the free curcumin or Cur-Nio as radio-sensitizing agents after 24 and 48 h. The Annexin V-FITC marker was used for the assessment of apoptosis, while the P.I. dye was applied for the evaluation of necrosis. The percentages of necrotic and apoptotic cells, along with statistical analysis obtained from the application of different doses and incubation periods of 48 and 72 h, are shown in Figures 6, 7. As shown in Figures 8A,B, irradiation caused apoptosis in 1.98 ± 0.06% and 2.56 ± 0.08% of cells when utilized at a dose of 1 Gy and 3 Gy for 24 h, respectively. Such percentages were 1.98 ± 0.06% and 2.11 ± 0.11% of cells irradiated with 1 Gy and 3 Gy for 48 and 72 h, respectively. The treatment of MCF-7 cells with the free curcumin (20 μg ml^−1^) caused apoptosis in 1.37 ± 0.12% and 1.32 ± 0.12% of cells irradiated with 1 Gy and 3 Gy at 24 h. Such percentages were reported to be 1.31 ± 0.07% and 1.45 ± 0.10% when the cells were exposed to Cur-Nio at 48 and 72 h, respectively. The combination therapy of cells with 20 μg ml^−1^ free curcumin and irradiation at a dose of 1 Gy showed 2.58 ± 0.08% and 2.55 ± 0.09% apoptosis rates at 24 h. The apoptosis rates at a dose of 3 Gy were reported to be 3.25 ± 0.09% and 3.55 ± 0.11% at 48 and 72 h, respectively. The apoptosis percentages of the combined treatment of cancer cells with irradiation and Cur-Nio at a dose of 1 Gy were reported to be 3.25 ± 0.10% and 3.11 ± 0.08%, while at a dose of 3 Gy, such percentages were 4.03 ± 0.05% and 4.11 ± 0.08% at 48 and 72 h, respectively (Figures 8A,B). The results illustrated that the combination of irradiation with radio-sensitizing agents significantly increased apoptosis in cancer cells compared with the single use of each treatment. In addition, the rate of apoptosis was significantly higher in cells exposed to the encapsulated form of curcumin than that in the free form. Moreover, as shown in Figures 8C,D, the necrosis ratios were almost 1.4 folds at 1 Gy and 1.2 fold at 3 Gy for 48 h. Such ratios were 1.3 fold at 1 Gy and 1.2 fold at 3 Gy for 72 h when the cells were treated by the combination of irradiation and nanoniosome-containing curcumin compared with cells exposed to irradiation alone. The necrosis ratios of cells exposed to irradiation and free curcumin were 1.2 folds at 1 Gy and 1.1 fold at 3 Gy for 48 h. Also, such ratios were 1.1 fold at 1 Gy and 1.0 fold at 3 Gy for 72 h. On the other hand, the necrosis ratios of cells treated with Cur-Nio and irradiation were almost 1.2 fold at 1 Gy and one fold at 3 Gy compared with those exposed to free curcumin for 48 h. These results displayed not only higher percentages of apoptosis and necrosis in cells exposed to the combination of irradiation and irradiation-sensitizing agents but also showed higher efficacy of irradiation doses (1 Gy vs. 3 Gy) and encapsulated form of curcumin in the induction of cell death in cancer cells.

## Discussion

The radioresistance of breast cancer cells leads to the neutralization of irradiation therapy as an adjuvant treatment. Studies demonstrated that curcumin is a radio-protectant agent with antioxidant activity and is known as a radio-sensitizing agent that damages the mitochondrial membrane and induces apoptosis in cancer cells (Chendil et al., 2004; Khafif et al., 2005; Girdhani et al., 2009). The main purpose of our study was to examine the effects of pre-treatment of curcumin-loaded PEGylated nanoniosome as a radiosensitizing agent, followed by irradiation on the MCF-7 cell line in comparison to free curcumin. First, we characterized the curcumin-loaded PEGylated nanoniosome using various techniques. The mean diameter of nanoparticles was almost 100 nm at the nanoscale size, which facilitates their entry to the blood barrier and increases their concentrations in cancer cells (Hemati et al., 2019b). The zeta potential value was reported to be negative, and there was an electrostatic repulsion between vesicles resulting in the stability and homogeneity of nanoniosomes (Chen et al., 2012; Spirou et al., 2018). Also, the accordance of AFM images with DLS results showed rounded and smooth shapes with rigid boundary nanostructures in the synthesized nanoniosomes. Hydrophilic-lipophilic balance (HLB) and phase transfer temperature are two essential parameters that affect entrapment efficiency. The higher transition temperature (Tc) of Tween 60 (55°C) and a lower HLB value (14.9) led to the highest entrapment efficiency of curcumin as a lipophilic drug (Palozza et al., 2006; Bnyan et al., 2018). Tween 60 has a high transfer temperature and an impermeable bilayer that reduces the release rate of curcumin and leads to a continuous release. Moreover, PEGylation of nanoniosomes improves their stability, enhances drug loading, reduces nanoparticle size, and decreases the drug-release rate in niosomal formulations (Haghiralsadat et al., 2017; Hemati et al., 2019a). The endocytosis mechanism of Cur-Nio plays a crucial role in penetrating and increasing the accumulation of curcumin into cells, causing a higher intensity in green and turquoise blue fluorescence. In other words, the nanoniosomal curcumin delivery system was more efficient in the delivery and localization of curcumin compared with the free one that crosses through the cell membrane by a diffusion process. These findings are in agreement with a number of studies conducted in this regard (Huang et al., 2014; Deljoo et al., 2019). Our results demonstrate that cells treated with irradiation produce intracellular reactive oxygen species (ROS), leading to a decreased proliferation rate (∼50% at 3 Gy) in cancer cells in a dose-dependent manner compared with control cells. We examined irradiation doses (1 Gy and 3 Gy) in combination with free curcumin and Cur-Nio based on the IC10 values. Our findings indicated that concomitant use of the free curcumin or niosome-containing curcumin with irradiation increased cell death by ∼1 and ∼1.5 folds in comparison to cells exposed to irradiation alone, respectively. Accordingly, our results were in compliance with studies performed in this regard (Girdhani et al., 2009; Swati et al., 2009; Goel & Aggarwal, 2010; Yallapu et al., 2010; Shehzad et al., 2013b; Minafra et al., 2019). We found that the combined use of curcumin with irradiation increased the efficiency of radiotherapy against breast cancer cells. The radio-sensitivity of curcumin is attributed to its influences on various molecules involved in the cellular signaling pathway, including PI3-kinase, NF-κB, STAT3, COX2, Akt, MAPK, AMPK, p53, Nrf2, Notch-1, β-catenin, and the upregulation of genes responsible for cell death that induce apoptosis and inhibit proliferation of cancer cell (Shehzad & Lee, 2013a). We also showed that the presence of curcumin-containing PEGylated nanoniosomes increased the radiation efficiency (3Gy) compared to free curcumin in breast cancer cells, and cell death changed from 50% to% 68% at 72 h. This phenomenon might be due to the fact that nanoniosomes promote solubility, stability, and bioavailability of curcumin and enhance the cellular uptake and localization of curcumin in cancer cells (Kolter et al., 2019; Karimi et al., 2020). Minafra et al. (2019) evaluated the radio-sensitizing ability of curcumin-containing solid nanoparticles (Cur-SLN) in comparison to free curcumin at three concentrations with increasing irradiation doses. They demonstrated that Cur-SLN caused higher radio-sensitivity in MCF7 cells by increasing curcumin bioavailability in a synergistic manner (Minafra et al., 2019). Ahmed et al. assessed the impact of free curcumin as a radiosensitizer on breast cancer cells before gamma irradiation. They showed that the combination of curcumin and irradiation increased cancer cell death by 62% compared to the single-use of curcumin, causing 48% apoptosis in cancer cells (Girdhani et al., 2009). Xu et al. (2018)sensitized colorectal cancer cells to ionizing irradiation by developing curcumin-based supramolecular nanofibers (Cur-SNF) and found these structures caused higher radio-sensitivity in these cancer cells compared to those treated with the free curcumin. These structures reduced tumor volume *in vivo* by inhibiting irradiation-induced nuclear factor kappa B activation (Xu et al., 2018). Our results were consistent with these results. Also, Liang et al. determined the cell survival effect of combined and single curcumin and cisplatin with irradiation on non-small cell lung cancer (NSCLC) A549 cells. The viability of these cells was reduced following treatment with curcumin, cisplatin, or curcumin + cisplatin with irradiation compared to a single dose of irradiation. These findings exhibited that curcumin and cisplatin promote radio-sensitivity of lung cancer cells through the inhibition of the epidermal growth factor receptor (EGFR)-associated signaling pathway (Cai et al., 2019). Their results were in line with our findings.

## Conclusion

In general, we have developed a new curcumin-containing PEGylated nanoniosome to enhance the radiosensitivity of breast cancer cells instead of using the free curcumin. The results demonstrated that PEGylated nanoniosomes have nanoscale size, spherical shape, high entrapment efficiency, and a controlled release pattern. Our study highlights the significance of the application of curcumin in sensitizing breast cancer cells to irradiation, and PEGylated nanoniosome is a very promising system for cancer therapy in combination with radiotherapy through boosting solubility, bioavailability, and cellular uptake of curcumin.

## Data Availability

The raw data supporting the conclusions of this article will be made available by the authors, without undue reservation.
